# Rat Mucosal Immunity following an Intensive Chronic Training and an Exhausting Exercise: Effect of Hesperidin Supplementation

**DOI:** 10.3390/nu15010133

**Published:** 2022-12-27

**Authors:** Patricia Ruiz-Iglesias, Sheila Estruel-Amades, Malén Massot-Cladera, Àngels Franch, Francisco J. Pérez-Cano, Margarida Castell

**Affiliations:** 1 Secció de Fisiologia, Departament de Bioquímica i Fisiologia, Facultat de Farmàcia i Ciències de l’Alimentació, Universitat de Barcelona (UB), 08028 Barcelona, Spain; 2 Institut de Recerca en Nutrició i Seguretat Alimentària (INSA-UB), UB, 08921 Santa Coloma de Gramenet, Spain; 3 Centro de Investigación Biomédica en Red de Fisiopatología de la Obesidad y la Nutrición (CIBEROBN), Instituto de Salud Carlos III, 28029 Madrid, Spain

**Keywords:** flavanone, flavonoid, IgA-coated bacteria, lymphocytes, mesenteric lymph node, microbiota, proliferation, secretory IgA

## Abstract

Stressful situations such as a high-intensity exercise or exhausting training programs can act as immune disruptors leading to transitory immunodepression status, which can be accompanied by alterations of the gastrointestinal functions. Hesperidin intake has demonstrated ergogenic activity and is able to influence the intestinal ecosystem and immunity. We aimed to investigate the effect of hesperidin consumption in rats submitted to an intense training and a final exhaustion test, focusing on the functionality of the intestinal immune system and on the cecal microbiota. Rats, supplemented or not with hesperidin, were intensively trained on a treadmill for 5 weeks. Samples were obtained 24 h after a regular training session, and immediately and 24 h after a final exhaustion test. Cecal microbiota and composition and function of mesenteric lymph node (MLN) lymphocytes and mucosal immunoglobulin A (IgA) were determined. Results showed that chronic intense exercise followed by an exhausting test induced changes in the intestinal immune compartment such as the distribution and function of MLN lymphocytes. Although the hesperidin supplementation did not prevent these alterations, it was able to enhance IgA synthesis in the intestinal compartment. This could be important in enhancing the immune intestinal barrier in this stressful situation.

## 1. Introduction

Immunonutrition focuses on the interface of nutrition and immunology and, in the last decades, it has become the focus of many studies. Within this field, physical exercise is a recent area of research that is fast developing due to the interest of athletes and also to the development of the nutrition market [1]. Nevertheless, high-intensity or exhausting training programs can cause stressful situations that can act as immune disruptors leading to a transitory immunodepression status. Consequently, elite endurance athletes have a higher frequency of upper respiratory tract infections following an intensive race such as a marathon or triathlon [2,3]. In particular, during and immediately after a high intensity exercise, a transient leukocytosis appears, which is later followed by leucopenia and the suppression of other immune players, producing a “window of opportunity” for pathogens [4,5]. Moreover, exhausting exercise changes the immunological profile, due to an increase in humoral immunity associated with a downregulated cellular immunity [4]. Therefore, vigorous exercise promotes the synthesis and release of T helper (Th)-2 (anti-inflammatory) cytokines in order to decrease the muscle damage resulting from inflammation, but increases susceptibility to infections [4,5,6,7,8]. Preclinical studies have demonstrated the alterations produced in systemic and intestinal lymphoid tissues after chronic and vigorous physical exercise. Thus, it has been reported that intensive training induces a decrease in the phagocytic activity, a differential pattern of cytokines, increased natural killer (NK) cytotoxic activity, and reduced lymphoproliferative activity [9,10].

Nevertheless, exercise does not only affect the immune system. Currently, the term of “exerkines” is used to define signaling moieties released in response to acute and/or chronic exercise that may affect a wide variety of organs [11]. The immune system, bone, nervous system, and cardiometabolic tissues (cardiovascular, adipose, endocrine systems, liver, and skeletal muscle) act as a source of exerkines and, at the same time, are directly affected by exercise [11]. Therefore, in addition to the disruption of the immune system, alterations of gastrointestinal functions can also appear. In fact athletes, especially endurance athletes, can suffer upper and lower gastrointestinal complaints, which can have negative effects on performance and also an impact on subsequent recovery [12,13]. In general, most gastrointestinal complaints are mild and include nausea, cramping, bloating, abdominal angina, and diarrhea [12,13], but mucosal erosions and ischemic colitis have also been observed after long-distance running [13]. The mechanisms underlying the prolonged exercise-induced gastrointestinal disorders likely include the redistribution of blood flow from the gut to the skin [12,13], but gut dysbiosis is also reported in some endurance runners [14,15]. In addition, preclinical studies have demonstrated that a high-intensity training reduces the salivary production of immunoglobulin (Ig) A, impairs the tight junction proteins’ gene expression, and modifies the mesenteric lymph nodes (MLNs) lymphocyte composition and function, increasing the ratio between Tαβ+ and B lymphocytes, reducing their proliferation capacity, and enhancing their interferon (IFN) γ secretion [16].

Hesperidin is a flavanone (hesperetin-7-rutinoside) found in citric fruits, such as mandarins, oranges, and lemons [17]. Many studies have demonstrated that hesperidin consumption offers beneficial health effects [18,19]. In this regard, in recent months, published reviews have summarized the effects of hesperidin in cancer [20,21,22], COVID-19 [23,24,25,26], Alzheimer’s disease [21], cardiovascular disease [25,27,28], alcoholic liver disease [29], inflammatory disease [30], diabetes [31], and rheumatoid arthritis [31], among others.

Previous studies have reported the influence of hesperidin intake in exercise both in clinical [32,33,34,35,36], and preclinical studies [37,38,39,40]. In particular, clinical studies demonstrated that a customized citrus flavonoid extract taken for 4 weeks increased cycling time-trial performance in male athletes [32] and enhanced anaerobic capacity and peak power during high intensity exercise in moderately trained men and women [36]. A longer study demonstrated that the intake of 2S-hesperidin for 8 weeks increased endogenous antioxidant capacity and performance after exhausting exercise in amateur cyclists [34,35]. Furthermore, preclinical studies showed that the administration of 200 mg/kg/3 times per week of hesperidin was able to prevent the overproduction of reactive oxygen species and the decrease in thymic and splenic antioxidant activities after an exhaustion test in trained rats [39]. Moreover, the flavanone enhanced NK cell cytotoxic and monocyte phagocytic functions, whereas it attenuated the secretion of cytokines by macrophages [40]. Similarly, the intake of 100 mg/kg hesperidin for 4 weeks before a continuous swimming exercise in rats improved the biochemical profile and antioxidant biomarkers [37].

On the other hand, hesperidin supplement was able to influence the intestinal ecosystem and immunity [41,42,43]. The administration of the flavanone for 4 weeks in healthy rats influenced the cecal microbiota, increasing the number of bacteria and particularly the proportion of *Lactobacillus* population, and also raised the intestinal IgA content [42]. In healthy humans, the consumption of orange juice for two months also produced an increase in *Lactobacillus* spp. abundance and in the acetic acid production [44]. In another study, the intake of two orange juices per day for seven days resulted in microbiota composition shifts, with an increase in the abundance of Clostridia operational taxonomic units [44]. Moreover, hesperidin intake in rats was able to change the lymphocyte composition in the MLNs, intestinal epithelium and the lamina propria [41,42]. In addition, hesperidin intake increased the IFN-γ secretion by lymphocytes of MLNs in immunized rats [41]. In a model of physical exercise, the administration of 200 mg/kg of hesperidin (3 times per week, for 5 weeks) enhanced the NK function and the proportion of phagocytic monocytes, attenuated the secretion of macrophage-derived cytokines and prevented the leukocytosis induced by exhaustion [40]. These results and the intestinal influence of hesperidin prompted us to study the influence of hesperidin intake during an intensive training and an exhausting exercise, on the intestinal immune system. Thus, the aim of the present study was to investigate the effect of hesperidin consumption during 5 weeks in rats submitted to an intense training and a final exhaustion test, focusing on the functionality of the intestinal immune system and on the cecal microbiota.

## 2. Materials and Methods

### 2.1. Animals, Exercise Training Program, and Hesperidin Supplementation

The animals and exercise training program were the same as previously reported [40]. Briefly, 3-week-old female Wistar rats (Envigo, Huntingdon, UK) were used. Female animals were chosen because they showed better adaptability and higher performance than male rats [9]. Animals were fed, ad libitum, with Teklad Global 14% Protein Rodent Maintenance Diet (Teklad, Madison, WI, USA). The animal procedure was approved by the Ethical Committee for Animal Experimentation of the University of Barcelona and the Catalonia Government (CEEA/UB ref. 464/16 and DAAM 9257, respectively), in full compliance with national legislation following the European Union Directive 2010/63/EU for the protection of animals used for scientific purposes.

Two specialized treadmills for rodents—a LE8700 treadmill (Panlab, Harvard Apparatus, MA, USA) and an Exer3/6 treadmill (Columbus, OH, USA)—were used. Firstly, after a 7-day acclimation period, all rats (4-week-old animals) were adapted to the treadmill (10 days with increasing time and speed). Afterwards, animals were distributed into four groups with similar ability to run: non-supplemented runner animals (RUN, *n* = 24), hesperidin-supplemented runner animals (H-RUN, *n* = 24), non-supplemented sedentary animals (SED, *n* = 8), and hesperidin-supplemented sedentary animals (H-SED, *n* = 8). H-RUN and H-SED groups received 200 mg/kg of body weight (BW) of hesperidin (HealthTech BioActives, Murcia, Spain) by oral gavage 3 times per week for 5 weeks. RUN and SED receive a matched volume of vehicle. RUN and H-RUN groups were then submitted to a 5-week intensive training in which, every Monday and Friday, rats carried out an exhaustion test, whereas on Tuesday, Wednesday, and Thursday rats ran for a period of time according to the maximum speed achieved in the previous Monday’s exhaustion test. At the end of the training program, each RUN and H-RUN groups were distributed into three subgroups to establish intestinal immunity at different time points. One subgroup (trained, T, and H-T groups, *n* = 8 each one) was euthanized 24 h after a regular training session. The other two subgroups carried out an additional final exhaustion test. One of these was euthanized immediately after carrying out an additional final exhaustion test (TE and H-TE groups, *n* = 8 each one), whereas the other one was euthanized 24 h after the final exhaustion test (TE24 and H-TE24 groups, *n* = 8 each one). Sedentary rats (both SED and H-SED groups) were euthanized randomly distributed over the three consecutive days. The experiment ended with 10-week-old animals.

### 2.2. Sample Collection and Processing

Rats were anesthetized with ketamine (90 mg/kg, Merial Laboratories S.A., Barcelona, Spain) and xylazine (10 mg/kg, Bayer A.G., Leverkusen, Germany) and exsanguinated. The MLNs, small intestine, submaxillary salivary glands (SMGs), and cecal content (CC) were collected.

Lymphocytes from MLNs were isolated by passing the tissue through a 40 µm sterile-mesh cell strainer (Thermo Fisher Scientific, Barcelona, Spain), as previously described [45].

The distal part of the small intestine was used to obtain gut wash (GW), as carried out previously [46]. Briefly, fecal content was removed from the intestine by flushing with cold phosphate buffered saline (PBS, pH 7.2), then the tissue was opened lengthwise, cut into 1–2 cm pieces, weighed, and incubated with PBS for 10 min in a shaker at 37 °C (55 shakings × min^−1^). After centrifugation (538× *g*, 4 °C, 10 min), supernatants were collected and stored at −20 °C until IgA quantification.

SMGs, CC, and fecal (from feces obtained at the moment of anesthesia) homogenates were obtained using a tissue homogenizer (for SMG, Polytron, Kinematica, Lucerne, Switzerland) or a Pellet Pestle Cordless Motor (for CC and fecal samples, Kimble, Meiningen, Germany), as described in previous studies [47], and kept at −20 °C until IgA quantification.

### 2.3. Cecal Microbiota Analysis by Fluorescence In Situ Hybridization Coupled to Flow Cytometry

The previous effects observed by the polyphenols interventions on particular bacteria groups, such as *Clostridium coccoides/Eubacterium rectale, Bifidobacterium* and *Lactobacillus/Enterococcus,* led us to evaluate the changes induced by exercise only on these populations. For that, sequencing was initially discarded and targeted fluorescence in situ hybridization coupled to flow cytometry (FISH-FCM) as previously used in our group [48], was used. Group-specific fluorochrome-conjugated probes (Erec482 5′-GCTTCTTAGTCARGTACCG, Bif164 5′-CATCCGGCATTACCACCC, and Lab158 5′-GGTATTAGCAYCTGTTTCCA) (Sigma-Aldrich, Madrid, Spain), which hybridize the bacterial 16S RNA of each particular group were used. Data were acquired by a FacsAria SORP sorter (BD Biosciences, San Diego, CA, USA) in the Flow Cytometry Unit (FCU) of the Scientific and Technological Centers of the University of Barcelona (CCiTUB) and the analysis was performed with FlowJo v.10 software (Tree Star Inc., Ashland, OR, USA). The results were normalized by total bacteria, which was detected adding propidium iodide (PI; Sigma-Aldrich) prior to the FCM acquisition. Commercial Flow Check^TM^ Fluorospheres (Beckman Coulter, Miami, FL, USA) were used to assess the total counts of bacteria.

### 2.4. Cecal Proportion of IgA-Coated Bacteria

The proportion of IgA-coated bacteria (IgA-CB) in CC was determined by FCM as previously described [48]. The CC homogenate was stained with rabbit anti-rat Ig polyclonal antibody conjugated to fluorescein isothiocyanate (FITC) (Abcam, Cambridge, UK). Bacteria were gated in a FacsAria SORP sorter (BD Biosciences) after PI staining (Sigma-Aldrich) and according to their forward (FSC) and side scatter (SSC) characteristics. Analysis was performed in the FCU of the CCiTUB using the FlowJo v.10 software.

### 2.5. Phenotypic Analysis of MLNs Lymphocytes

Lymphocytes from MLNs were extracellularly and intracellularly stained as previously reported [16]. Mouse anti-rat monoclonal antibodies (mAb) conjugated to fluorochromes—fluorescein isothiocyanate (FITC), phycoerythrin (PE), peridinin chlorophyll protein (PerCP), allophycocyanin (APC) or brilliant violet 421 (BV421): FITC-TCRαβ, FITC-CD8β, FITC-CD25, PE-CD161a, PE-TCRγδ, PE-CD4, PerCP-CD8α, APC-CD4, and BV421-CD45RA (BD Biosciences) and APC-FoxP3 (eBioscience, Frankfurt, Germany). For extracellular staining, cells were incubated with saturating amounts of mAb in PBS containing 2% Fetal Bovine Serum (FBS) and 0.1% NaN_3_. For intracellular staining, cells were previously extracellularly labeled with anti-CD4-PE and anti-CD25-FITC mAb, then treated with Foxp3 fixation/permeabilization kit (eBioscience) and finally intracellularly stained with anti-Foxp3-APC mAb. Cells were fixed with 0.5% p-formaldehyde and stored at 4 °C in darkness until analysis by FCM. A negative control staining without any mAb and a staining control for each mAb were included.

Analyses were performed using a Gallios Cytometer (Beckman Coulter, Miami, FL, USA) in the FCU of CCiT-UB and by Flowjo v10 software.

### 2.6. Proliferative Activity of Lymphocytes from MLNs

Cells (10^5^ cells/well) were incubated in quadruplicate in 96-well plates (TPP, Sigma-Aldrich) and stimulated or not with concanavalin (Con) A (5 µg × mL^−1^, Sigma-Aldrich) for 48 h as previously described [16]. Cell proliferation was quantified using a BrdU Cell Proliferation Assay kit (MerckMillipore, Darmstadt, Germany), according to manufacturer’s instructions. The proliferation rate was calculated by dividing the optical density of ConA stimulated cells with the optical density of non-stimulated cells.

### 2.7. IgA Quantification

The concentrations of IgA in GW, SMGs, and in CC and fecal homogenates were quantified by a sandwich ELISA (Bethyl Laboratories Inc., Montgomery, AL, USA) as previously described [49]. The IgA content in SMGs and CC was normalized by total protein concentration which was measured using the Pierce^®^ 660 nm Protein Assay Reagent (Thermo Fisher Scientific) following the manufacturer’s instructions.

### 2.8. Statistical Analysis

Data were statistically analyzed by IBM Social Sciences Software Program (SPSS, version 26.0, Chicago, IL, USA) and Rstudio v4.04 (Rstudio, Inc., Boston, MA, USA) with R version 3.6.1 (R Core Team 2021, R Foundation for Statistical Computing, Vienna, Austria). The normality and homoscedasticity of the data were tested by Shapiro–Wilk’s and Levene’s test, respectively. In this case, we applied a two-way ANOVA test and, if significant differences were found, Tukey’s post hoc test was carried out. Non-parametric Aligned Rank Transform for non-parametric factorial ANOVA (ART-ANOVA) followed by emmeans post hoc (Tukey-adjusted *p* value) were applied, using the ARTool [45,46] and emmeans [47] packages, respectively, for Rstudio.

When significant differences were detected (*p* ≤ 0.05), the *p* values obtained in the two-way ANOVA or the ART-ANOVA for the variables exercise (E), hesperidin supplementation (H), and the interaction between them (E × H) are indicated in bold in the legend box. Changes due to the exercise are represented in the figure using different letters above the bars. When the E × H interaction was significant, changes between particular groups were represented with an asterisk above the respective bars.

## 3. Results

### 3.1. Changes in Morphometric Variables

**Figure 1:**
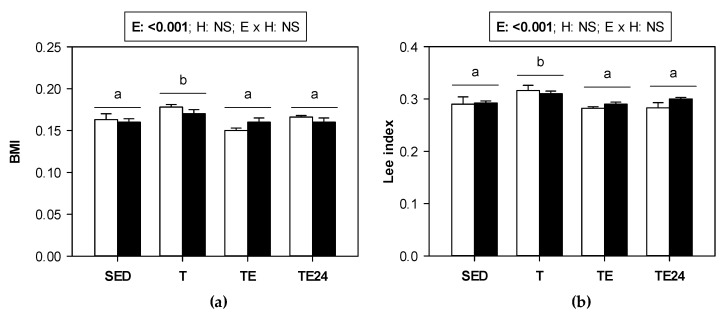
(**a**) Body mass index (BMI) and (**b**) Lee index in sedentary (SED) and runner rats (training for five weeks) that were classified into T group, with values obtained 24 h after a regular training session, TE group, with values obtained immediately after a final exhaustion test, and TE24 group, with values obtained 24 h after the final exhaustion test. The non-supplemented groups are represented by white bars and the hesperidin-supplemented groups by black bars. E = exercise; H: hesperidin supplementation; E × H = interaction between E and H; NS = no significant differences. Data are expressed as mean ± SEM (*n* = 8). Different letters between groups indicate significant differences (*p* < 0.05) due to exercise.

The training program, both in non-supplemented animals and those supplemented with hesperidin, induced a slight but significant increase in the body mass index (BMI) and Lee index (Figure 1a,b, respectively). The final exhaustion test reduced both BMI and Lee index with respect to the values present in trained rats (TE and TE24 groups vs. T group). Such a decrease must be attributed to the weight lost (mainly water) due to exhaustion. BMI and Lee index did not change due to hesperidin supplementation.

### 3.2. Changes in Cecal Microbiota

**Figure 2:**
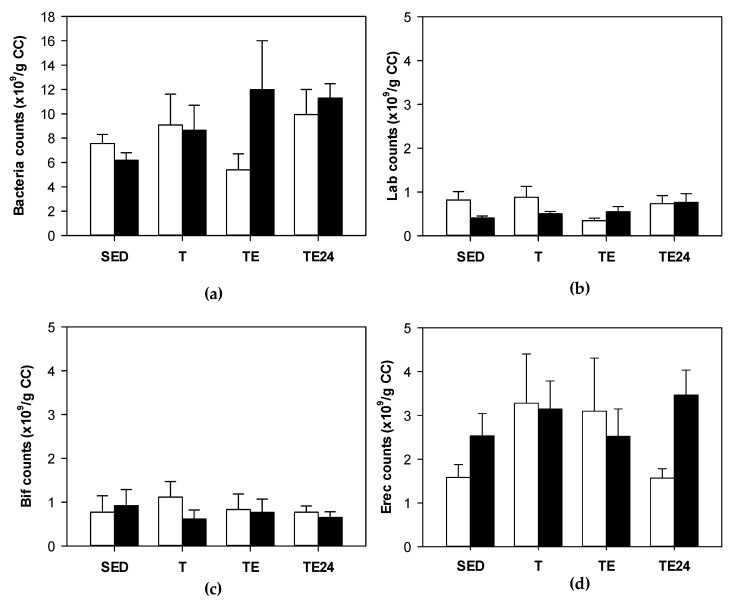
Total bacteria (counts ×10^9^/g cecal content) (**a**); *Lactobacillus* (counts ×10^9^/g cecal content) (**b**); *Bifidobacterium* (counts ×10^9^/g cecal content) (**c**); *Clostridium coccoides/Eubacterium rectale* (counts ×10^9^/g cecal content) (**d**). The non-supplemented groups are represented by white bars and the hesperidin-supplemented groups by black bars. CC = cecal content; SED = sedentary groups; T = trained groups; TE = T groups with an additional exhaustion test; TE24 = TE groups 24 h after the exhaustion test. Data are expressed as mean ± SEM (*n* = 8).

The content of total bacteria in the cecum was established in sedentary and runner rats from non-supplemented groups and hesperidin-supplemented groups (Figure 2a). Neither exercise nor hesperidin supplementation significantly changed the total content of cecal bacteria. Likewise, we did not find significant variations in the abundance of *Lactobacillus*, *Bifidobacterium* and *Clostridium coccoides/Eubacterium rectale* groups in cecum due to hesperidin supplement or exercise (Figure 2b–d).

### 3.3. Changes in the Number of IgA-Coated Bacteria

**Figure 3:**
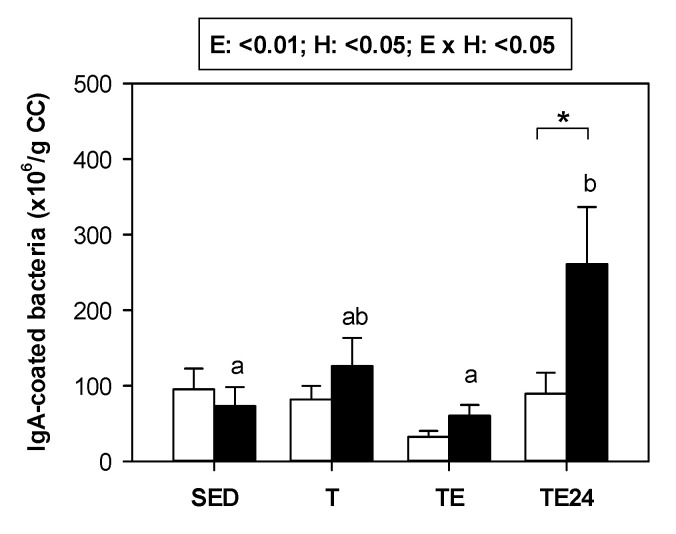
Number of cecal bacteria coated to IgA (×10^6^/g cecal content). The non-supplemented groups are represented by white bars and the hesperidin-supplemented groups by black bars. CC = cecal content; SED = sedentary groups; T = trained groups; TE = T groups with an additional exhaustion test; TE24 = TE groups 24 h after the exhaustion test. E = exercise; H: hesperidin supplementation; E × H = interaction between E and H. Data are expressed as mean ± SEM (*n* = 8). As E × H interaction was significant, Tukey’s post hoc test was carried out, and changes between particular groups are represented with different letters above those groups that showed different results, and changes due to hesperidin supplementation are represented with an asterisk.

Overall, the amount of cecal bacteria coated to IgA did not change exclusively due to exercise or hesperidin supplementation (Figure 3). Nevertheless, in those animals supplemented with hesperidin, there was an increase in the proportion of bacteria coated to IgA 24 h after carrying out the final exhaustion test. In these animals, the number of IgA-coated bacteria was almost three-fold higher than that in the non-supplemented group.

### 3.4. Lymphocyte Composition of MLNs

**Figure 4:**
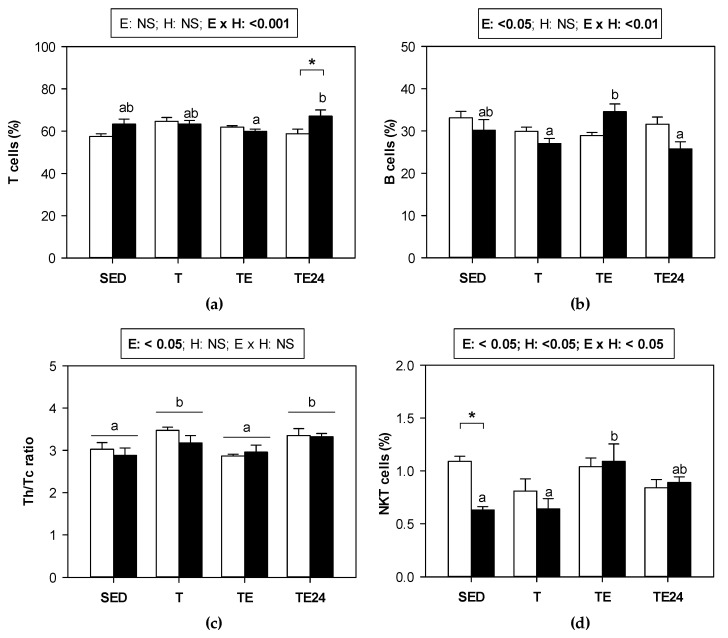
Proportion of the main lymphocyte populations in MLNs: (**a**) T cells, (**b**) B cells, (**c**) Th/Tc ratio, and (**d**) NKT cells. The non-supplemented groups are represented by white bars and the hesperidin-supplemented groups by black bars. SED = sedentary groups; T = trained groups; TE = T groups with an additional exhaustion test; TE24 = TE groups 24 h after the exhaustion test. E = exercise; H: hesperidin supplementation; E × H = interaction between E and H; NS = no significant differences. Data are expressed as mean ± SEM (*n* = 8). When E × H interaction was significant, Tukey’s post hoc test was carried out, and changes between particular groups are represented with different letters above those groups that showed different results, and changes due to hesperidin supplementation are represented with an asterisk.

Figure 4 summarizes the proportion of the main lymphocyte subsets found in MLNs of sedentary and runner animals that were supplemented or not with hesperidin. As we can see, in non-supplemented animals, exercise did not modify the proportion of T and B lymphocytes. However, in those groups that received hesperidin, T cell proportion increased by 14%, and B cell proportion diminished 24 h after exhaustion with respect to values obtained immediately after the exhaustion test (Figure 4a,b). When considering the ratio between Th and cytotoxic (Tc) cells in MLNs, an increase in the ratio was observed in trained animals, regardless of hesperidin supplementation. However, immediately after the final session of exhausting exercise, the ratio was lower and, 24 h later, it was recovered (Figure 4c).

The minor population of NKT cells, which represents about 1% of T cells, did undergo some changes due to exercise just in the hesperidin-supplemented animals. There was an increase in the percentage of NKT cells after exhaustion in hesperidin-supplemented animals, which showed a lower proportion of these cells than the non-supplemented group in the sedentary condition (about 60% of that of non-supplemented group) (Figure 4d).

The phenotype of activated and regulatory T cells was also considered in the Th lymphocytes of MLNs. No changes due to exercise or hesperidin supplement were observed in these two subsets (Table 1).

**Figure 5:**
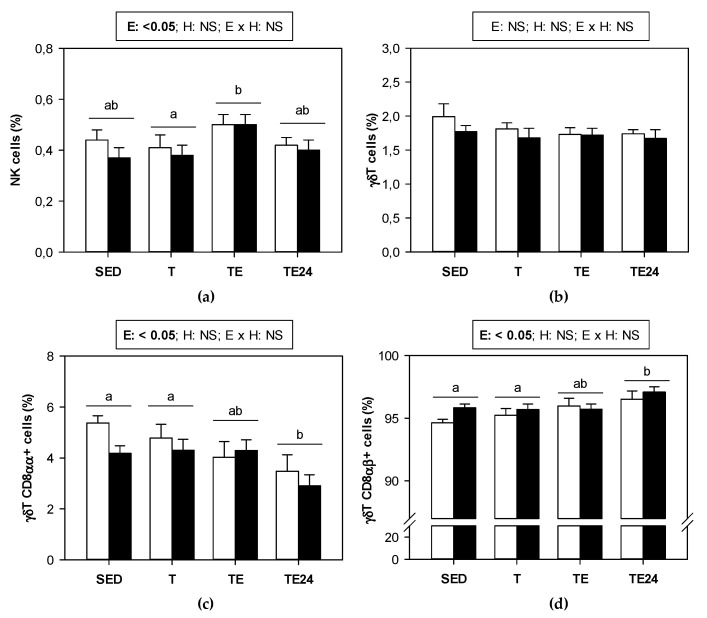
Proportion of the minor lymphocyte populations in mesenteric lymph nodes: (**a**) NK cells, (**b**) γδT cells, (**c**) γδT CD8αα+ cells, and (**d**) γδT CD8αβ+ cells. The non-supplemented groups are represented by white bars and the hesperidin-supplemented groups by black bars. SED = sedentary groups; T = trained groups; TE = T groups with an additional exhaustion test; TE24 = TE groups 24 h after the exhaustion test. E = exercise; H: hesperidin supplementation; E × H = interaction between E and H; NS = no significant differences. Data are expressed as mean ± SEM (*n* = 8). Statistical analysis (ART-ANOVA test): Different letters indicate significant differences due to exercise.

Figure 5 summarizes the proportion of the minor lymphocyte subsets found in MLNs of sedentary and runner animals that were supplemented or not with hesperidin. NK cell proportion underwent an increase just after exhaustion in both non-supplemented and hesperidin-supplemented animals (Figure 5a). On the other hand, although the proportion of γδT lymphocytes did not vary by either exercise or hesperidin supplementation in MLNs (Figure 5b), when considering the distribution between CD8αα+ and CD8αβ+ cell subsets, some changes associated with exercise appeared. In particular, we observed that 24 h after the final exhaustion test, the proportion of γδT CD8αα+ cells decreased whereas that of γδT CD8αβ+ cells increased (Figure 5c,d).

### 3.5. Lymphoproliferative Activity of MLNs

**Figure 6:**
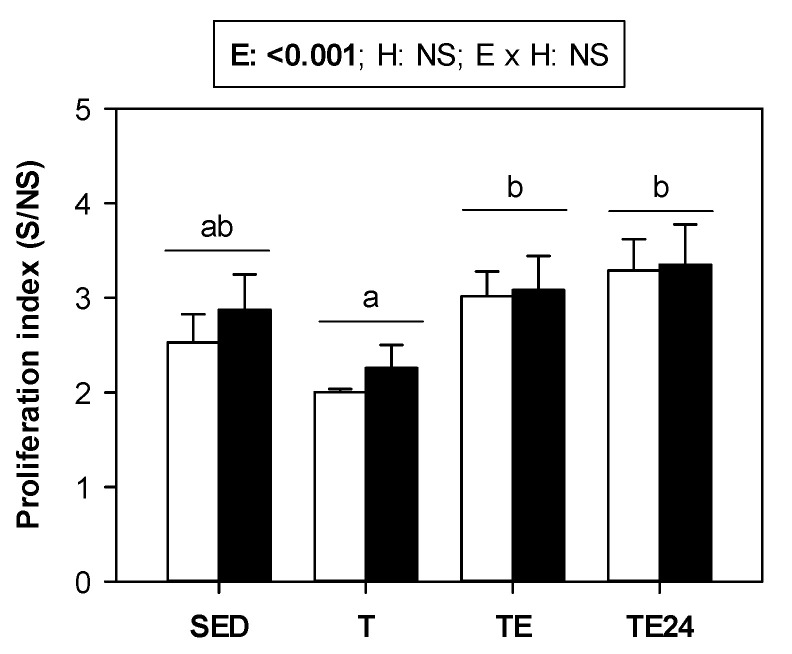
Proliferative activity of MLNs. The non-supplemented groups are represented by white bars and the hesperidin-supplemented groups by black bars. SED = sedentary groups; T = trained groups; TE = T groups with an additional exhaustion test; TE24 = TE groups 24 h after the exhaustion test. E = exercise; H: hesperidin supplementation; E × H = interaction between E and H; NS = no significant differences. Data are expressed as mean ± SEM (*n* = 8). Statistical analysis (ART-ANOVA test): Different letters indicate significant differences due to exercise.

The proliferative activity of lymphocytes from MLNs was established (Figure 6). Training for 5 weeks tended to decrease their proliferative activity in both non-supplemented and hesperidin-supplemented groups. After the final exhaustion test, the lymphoproliferative activity increased.

### 3.6. Changes in Mucosal IgA

**Figure 7:**
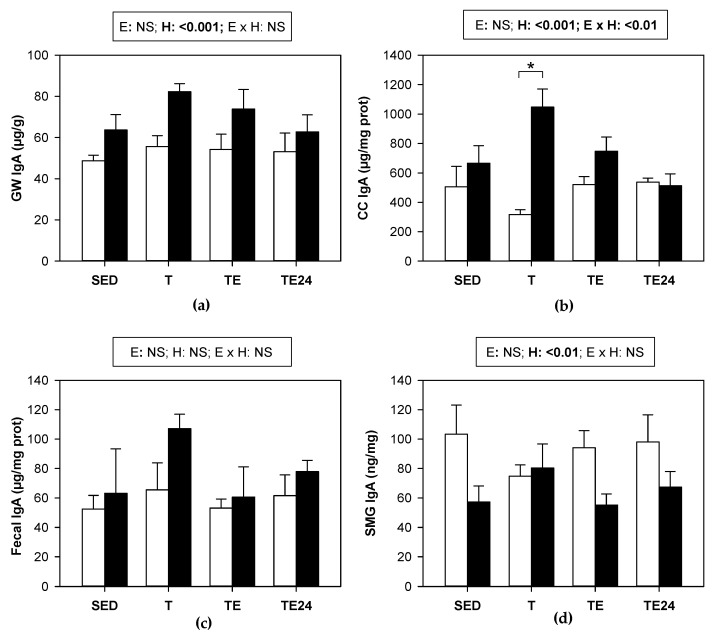
IgA concentration in: (**a**) gut wash; (**b**) cecal content; (**c**) fecal homogenate; (**d**) submaxillary glands. The non-supplemented groups are represented by white bars and the hesperidin-supplemented groups by black bars. GW = gut wash; CC = cecal content; SMG = submaxillary glands; SED = sedentary groups; T = trained groups; TE = T groups with an additional exhaustion test; TE24 = TE groups 24 h after the exhaustion test; E = exercise; H: hesperidin supplementation; E × H = interaction between E and H; NS = no significant differences. Data are expressed as mean ± SEM (*n* = 8). Statistical analysis (ART-ANOVA test): global effect of hesperidin supplementation is indicated in the corresponding box above. When E × H interaction was significant, an emmeans post hoc test (Tukey-adjusted *p* value for non-parametric data) was carried out, changes due to hesperidin supplementation are represented with an asterisk.

In the intestinal compartment, training or the final exhaustion test did not modify the IgA content in either the GW, cecal content, or feces (Figure 7a–c, respectively). In contrast, hesperidin supplementation did produce, overall, an increase in the IgA concentration found in small intestine and the cecum. In fact, in this last compartment, trained rats showed a three-fold increase in IgA content due to hesperidin supplementation. No significant changes were observed in feces (Figure 7c).

We also studied the IgA content in the SMGs. The IgA concentration was not significantly modified by training or final exhaustion test. Nevertheless, in general, supplementation with hesperidin decreased the IgA content in this mucosal compartment in most of the studied situations (Figure 7d).

## 4. Discussion

Although moderate physical exercise is good for the prevention of many chronic diseases, prolonged intense exercise may have negative health consequences, many of which may be mediated by physiological pathways activated by chronic stress. In consequence, among other effects, chronic exposure to stress exerts detrimental effects on immune function [4,50] and also the gastrointestinal ecosystem [13]. In these situations, nutritional supplements, such as polyphenol intake [51,52], could be useful for attenuating such conditions. Here we assessed the effect of hesperidin supplementation on the functionality of the intestinal immune system and the cecal microbiota of rats submitted to an intense training and a final exhaustion test. Previously, we have demonstrated that hesperidin supplementation during training partially improved exercise performance, enhanced the innate immunity by means of increasing the NK cell function as well as the proportion of phagocytic cells, and attenuated the release of some cytokines by macrophages. Moreover, hesperidin prevented the leukocytosis induced by exhaustion and promoted a higher proportion of Th cells in the thymus, blood, and spleen, 24 h after the exhaustion test [40].

Firstly, we focused on the gut microbiota which, in addition to its role in nutrient availability and vitamin synthesis, forms an intestinal barrier that helps to prevent pathogen colonization [53]. In addition, in the last decade, there has been an exponentially growing interest in the gut−brain axis, suggesting that microbiota has modulatory roles in neurodevelopment, brain function, and neurodegenerative diseases, and environmental factors such as diet and stress could modify this axis in a bidirectional pathway [54]. Although some researchers showed changes in the intestinal microbiota composition due to intense exercise [15,55,56], mostly studied by sequencing techniques, we could not detect alterations in the targeted bacteria considered in any of the three exercise conditions studied. The lack of significant changes in the gut microbiota could be due to the technique used (FISH-FCM) with low sensitivity and applied to only a few bacterial groups. In further studies, it would be necessary to apply a more sensitive assay, such as 16S rRNA sequencing methodology, that will provide more sensitive values and a wider range of information or even the Shotgun sequencing in order to obtain information regarding functionality in addition to the taxonomic data. On the other hand, we found that hesperidin supplementation did not significantly affect cecal microbiota in either sedentary or runner rats. These results agree with those reported by Unno et al. [57] and with an in vitro study showing that hesperidin had no effect on *Lactobacillus* spp. cultures [58]. However, in healthy humans, the consumption of orange juice for two months produced an increase in *Lactobacillus* spp. [44], and the intake of orange juice for 60 days increased the abundance of *Bifidobacteriaceae* [59]. In addition, a previous study in Lewis rats using a similar dosage of hesperidin increased the total bacteria counts and also the amount of *Bifidobacterium* and *Lactobacillus/Enterococcus* [42] but had no effect on the *C.coccoides/E.rectale* abundance in the cecum content of rats. The differences found for *Bifidobacterium* and *Lactobacillus/Enterococcus* bacteria could be partially due to the different rat strain (Lewis vs. Wistar) that will induce a different sensitivity to the flavanone intake.

Beyond gut microbiota, the intestinal immune system contains MLNs, where lymphocytes come into contact with dendritic cells charged with intestinal pathogens and will be easily mobilized to the blood. We found that training for 5 weeks did not significantly modify the proportion of total T cell population but increased the Th/Tc cell ratio in MLNs, which was normalized immediately after exhaustion, in agreement with previous results in this compartment [16] as well as in the spleen [10]. It is well known that exhaustion mobilizes T lymphocytes, and from the decrease in Th/Tc proportion in the TE group with respect to T group, it could be suggested that Th lymphocytes will be mobilized faster than Tc cells. The mobilization of Th cells from MLNs or spleen will increase blood Th cell proportion as previously reported in rats similarly trained and exhausted [10]. Later on, lymphopenia occurs due to movement back to the lymphoid organs, which could be responsible for the increase in the Th/Tc cell ratio in MLNs 24 h after exhaustion. In rats receiving hesperidin supplement, we did not find differences in the Th/Tc cell ratio with compared to non-supplemented animals but there was a higher T cell proportion and a lower B cell proportion 24 h after exhaustion. A higher T cell proportion by hesperidin has also been reported in blood from similarly trained and exhausted animals [40]. These increases in T cell proportions in MLNs and blood of rats that received hesperidin agree with the effect reported in the thymus, where it seems that the flavanone enhanced T cell maturation [40]. Likewise, it has been reported that, in non-trained rats, hesperidin supplement did increase T cell proportion (both Th and Tc lymphocytes) in MLNs [41,42].

On the other hand, the study of activated Th or regulatory T cells in MLNs revealed no changes due to exercise or hesperidin. However, the proportion of Tγδ cells with the phenotype CD8αα+ decreased 24 h after the final exhaustion, which was not prevented by hesperidin supplementation. Similarly, in both non-supplemented and hesperidin-supplemented animals, NK cell proportion increased immediately after exhaustion, which could be due to Th cell mobilization, decreasing the percentage of Th cells and reciprocally increasing other lymphocyte proportions. Lastly, the NKT cell proportion in sedentary rats was decreased by hesperidin supplementation, but those animals that received the flavanone, increased NKT cell proportion just after exhaustion, which could be a consequence of T cell mobilization in these animals.

The function of MLNs T lymphocytes, established by their proliferative ability, tended to decrease by chronic and intensive training. However, the final exhaustion, even 24 h later, increased their function, which is in agreement with results reported in similarly trained and exhausted animals [16], although other researchers reported a reduced T-cell proliferation [60]. Hesperidin supplementation did not change the results in terms of lymphocyte proliferation. In fact, previous results in non-trained animals treated with a similar dosage of hesperidin showed that it does not affect MLNs lymphocyte proliferative response or the release of cytokines from these cells [42].

In addition to the gut microbiota and the MLNs lymphocytes, one of the main players of the mucosal immune system is secretory IgA. This antibody plays an important role in the intestinal surface where it neutralizes toxins and viruses, blocks the excessive bacterial adherence or translocation, clears unwanted macromolecular structures at the epithelial surface, and directs sampling of luminal antigen [61]. Very prolonged bouts of exercise have been associated with a decreased secretory IgA production [62], which could explain, at least in part, the higher susceptibility to mucosal infections observed in athletes [63,64]. Here we found that, in the intestinal compartment, the applied intensive exercise conditions did not modify the IgA content, in agreement with a previous study [16], although in this last case, a decreased salivary IgA concentration was observed. Although exercise was not able to significantly modify mucosal IgA, hesperidin supplementation was able to enhance IgA concentration in the small intestine and cecal content, which was observed the most in chronically trained group (H-T group). In agreement, a similar hesperidin supplementation in nonrunner rats showed higher small IgA content in the intestine but not in the cecum [42], and the intake of a diet containing 0.5% hesperidin also increased fecal IgA concentration [41]. Likewise, it has been suggested that flavonoids such as hesperidin could increase mucosal IgA, among other immune-enhancing effects that could help in the treatment of COVID-19 [23].

The fact that 24 h after exhaustion, rats treated with hesperidin raised the amount of IgA-coated bacteria in the cecum is in agreement with the influence of hesperidin increasing IgA levels in the small intestine and the cecal content reported here and, previously, in cecum and feces [41,42]. This effect had already been observed in nonexercised animals [42], and could reflect the protective role of the flavanone in the mucosal immune system that appeared hours after exhaustion. Therefore, given this hesperidin intake consequence along with its ergogenic effect, it could be important to consider its supplementation in stressful situations, such as in some diseases, gastrointestinal complaints, and exhausting exercise.

## 5. Conclusions

Chronic intense exercise followed by an exhausting test in rats induces changes in the intestinal immune compartment as observed in the distribution and function of lymphocytes from mesenteric lymph nodes. Although the hesperidin supplementation applied here did not prevent the main alterations in lymphocyte subsets, it was able to enhance IgA synthesis in the intestinal compartment, which could counteract the immune and gastrointestinal barrier alterations induced by intense and exhausting exercise.

## Figures and Tables

**Table 1:** Proportion of the activated and regulatory Th cells in MLNs.

Cells	Diet	SED (%)	T (%)	TE (%)	TE24 (%)
CD4+CD25+ cell proportion in Th cells	non-S ^1^	2.19 ± 0.183	2.16 ± 0.167	2.39 ± 0.222	2.05 ± 0.149
	Hesp ^2^	2.27 ± 0.117	2.06 ± 0.217	2.52 ± 0.282	2.27 ± 0.131
CD4+CD25+ Foxp3+ cell proportion in Th cells	non-S	1.79 ± 0.110	2.00 ± 0.1061	1.65 ± 0.260	1.75 ± 0.200
	Hesp	2.06 ± 0.114	1.93 ± 0.158	1.78 ± 0.310	1.98 ± 0.119

^1^ non-S = non-supplemented group, ^2^ Hesp = hesperidin-supplemented group.

## Data Availability

Data are contained within the article.
